# A large EEG dataset for studying cross-session variability in motor imagery brain-computer interface

**DOI:** 10.1038/s41597-022-01647-1

**Published:** 2022-09-01

**Authors:** Jun Ma, Banghua Yang, Wenzheng Qiu, Yunzhe Li, Shouwei Gao, Xinxing Xia

**Affiliations:** 1 School of Mechatronic Engineering and Automation, School of Medicine, Research Center of Brain-Computer Engineering, Shanghai University, Shanghai, China; 2 Engineering Research Center of Traditional Chinese Medicine Intelligent Rehabilitation, Ministry of Education, Shanghai, China

**Keywords:** Neural decoding, Data processing

## Abstract

In building a practical and robust brain-computer interface (BCI), the classification of motor imagery (MI) from electroencephalography (EEG) across multiple days is a long-standing challenge due to the large variability of the EEG signals. We collected a large dataset of MI from 5 different days with 25 subjects, the first open-access dataset to address BCI issues across 5 different days with a large number of subjects. The dataset includes 5 session data from 5 different days (2–3 days apart) for each subject. Each session contains 100 trials of left-hand and right-hand MI. In this report, we provide the benchmarking classification accuracy for three conditions, namely, within-session classification (WS), cross-session classification (CS), and cross-session adaptation (CSA), with subject-specific models. WS achieves an average classification accuracy of up to 68.8%, while CS degrades the accuracy to 53.7% due to the cross-session variability. However, by adaptation, CSA improves the accuracy to 78.9%. We anticipate this new dataset will significantly push further progress in MI BCI research in addressing the cross-session and cross-subject challenge.

## Abstract (summary)

**Table:** 

Measurement(s)	Electroencephalography
Technology Type(s)	motor imagery

## Background & Summary

Motor imagery-based brain-computer interface (MI-BCI), where in participant performs a mental rehearsal of a particular motor movement is an investigated protocol. Compared with other brain-computer interface (BCI) paradigms, MI-BCI can provide users with direct communication without limb movement or external stimulation. Although the study of splitting the same session data for training and testing has been relatively mature, approaches of cross-session and cross-subjects are limited. BCI competitions^1^, BCI2000 dataset^2^, societies^3^, and journal publications^4–6^ provide free motor imagery (MI) datasets and help researchers improve algorithms in the same session and subject, especially for the BCI competition dataset^7–11^. However, only containing one or two sessions in these datasets cannot meet the requirements of cross-session modeling. Hence, owing to the great significance of cross-session and cross-subjects, a dataset containing multiple independent session data for the same subject is proposed to reduce the training data in the test session.

In this paper, we recorded MI-BCI Electroencephalogram (EEG) dataset simultaneously with 5 independent sessions from 25 subjects. Subjects conduct MI experiments every 2 or 3 days, and 100 trials per experiment are integrated as a session. We validated our datasets using time-domain, spatial, and classification analysis. The dataset supports in-depth study of parameters optimization^12^, electrooculogram artifact denoising^13^, brain network^14,15^, and neuroimaging^16^. The dataset is stored on the link provided by figshare^17^.

We provide some classic MI algorithms and deep learning algorithms such as common spatial patterns (CSP)^18^ filter bank common spatial pattern (FBCSP)^19^, filter-bank convolutional network (FBCNet)^20^, EEGNet^21^ deep convolutional network (deep ConvNets)^22^, and adaptive transfer learning^23^. These algorithms are used in within-session training and cross-session training. Cross-session modeling uses different sessions for training and testing separately while within-session modeling is based on the same session. Therefore, cross-session modeling is more valuable than within-session. The swallow and tongue bulge MI tasks are designed to study cross-session training^24^. The results show that the cross-session training model in the same subject has certain distinguishability. However, transfer learning^25^ has limited cross-session training results. The adaptive transfer learning methods proposed by^18,26,27^ use a small amount of target session data to improve classification accuracy. We provide five separate sessions of each subject in the dataset that comply with cross-session training research. Finally, we introduce analysis of variance (ANOVA) to compare the classification accuracy of various conditions with chance level and CSP to verify the dataset’s quality.

This paper presents EEG data from 25 subject for 5 independent sessions of left-hand and right-hand grasping MI tasks, containing 12,500 (=5*25*100) trials. Subjects are asked to repeatedly imagine movements based on cues while their EEG data are recorded. During the experiment, subjects are kept quiet and supervised by the experimenter to ensure the reliability of the collected data. The public dataset includes preprocessed and experimental data that can be directly used for classification so that researchers can use the data directly.

We provide the benchmarking classification accuracy for three conditions, namely, within-session classification (WS), cross-session classification (CS), and cross-session adaptation (CSA), using various machine learning algorithms, including deep learning, with subject-specific models. Findings, WS achieves an average classification accuracy of up to 68.8%, while CS degrades the accuracy to 53.7% due to the cross-session variability. However, by adaptation, CSA improves the accuracy to 78.9%. Compared with the chance level, ANOVA statistical results show that both WS and CSA have significant differences (P < 0.001). However, there is no significant difference between CS and chance level (P > 0.05). The main reason is the difficulty of cross-session modeling. All classification accuracies of three conditions are provided in Supplementary Tables.

The classification performance of WS and CSA reflects the quality of the dataset. In particular, for CSA, significant performance improvement can be seen from the results, which affirms the feasibility of adaptation techniques in the cross-session condition. However, some accuracies of the WS and CSA are still below the chance level. EEG signals constantly change, and data from the same session on the same day can vary greatly from trial to trial. EEG signal of different brain states is more prone to misclassification, which makes the average classification accuracy less than 50%. Different brain states cause a lot of confusion in the features of different classes. In the results of CSP and FBCSP, the co-variance shift between training set and validation set for the same task is significant, which is a common observation in BCI. So misclassification can happen when you have a model but the test set is shifted away from the model’s condition and the classification accuracy will be below 50%. Even though the model shows high classification accuracy in the training set, there is a strong uncertainty in the classification results after the brain state shift away.

The CS exhibits poor classification performance. Since there is no method specifically designed for the CS, the benchmark method of WS is used for verification. Nonetheless, high classification performance is still found among a few subjects, indicating that this dataset has the potential for cross-session modeling. Cross-subject MI modeling can address the need for each modeling session for rehabilitation training of stroke patients and enhance the usability of stroke rehabilitation training. We anticipate this new dataset will significantly push further progress in MI-BCI research in addressing the cross-session and cross-subject challenge.

## Methods

### Subjects

Twenty-five healthy subjects (age 20–24, 12 females) without MI-based BCI experience were recruited for the experiment. At the beginning of the experiment, each subject signed a “Notice of Experimental Intention and Experimental Consent” to ensure their rights and interests. All the subjects were paid after the experiment. The participants were identified only by their aliases “sub-001” through “sub-025”. The study was approved by Shanghai second Rehabilitation Hospital Ethics Committee (approval number: ECSHSRH 2018-0101) and was in accordance with the Declaration of Helsinki.

### Experimental paradigm

**Fig. 1:**
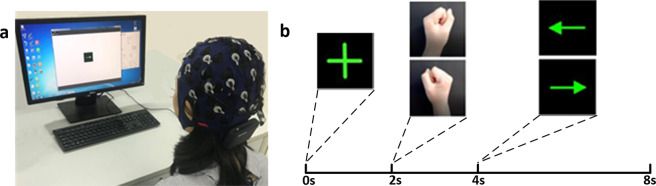
Data acquisition scenario. Informed consent was obtained from the individual in the figure for the publication of the images. (**a**) The experimental scene, (**b**) Motor imagery experiment procedure.

Before the experiment, each subject was explained the experimental method and steps, and all subjects had a full understanding of the whole process. Meanwhile, the experimenter was responsible for supervising the experimental process to ensure reliability. The experiment was carried out in a spacious and closed laboratory. Subjects sat on a chair one meter away from the 15-inch LCD monitor, as shown in the Fig. 1a. As shown in Fig. 1b, each trial started with a fixation cross in the center of the monitor to remind the subjects to pay attention to the upcoming task. When a left-handed or right-handed movement appeared on the monitor, the subjects were reminded of the next movement to imagine. The subjects began to repeatedly imagine the left-hand or right-hand grasping (kinetic motor imagery) when the left or right arrow appeared on the monitor. The duration of each trial was 7.5 s, each session contained 100 trials, and there were 4 break times during the experiment. They imagined the movement according to the video and audio cues. To maintain the physical and mental condition of the participants and high signal quality, the subjects took sufficient breaks and kept static as much as possible. The experimental process was shown in Table 1, including subjects’ reading the notice, equipment wearing and debugging, MI experiment and inspection data, etc.

**Table 1:** Experimental procedures.

Experimental procedure	Required time (min)	Cumulative time (min)
Fill in the questionnaire	5	5
Wear acquisition EEG equipment	25	30
Debug the signal	5	35
Motor imagery Experiment	35	70
Verify data	2	72

The EEG signal was recorded completely in the experiment, and the rest time can be adjusted according to the subject’s status.

### Data collection and preprocessing

**Fig. 2:**
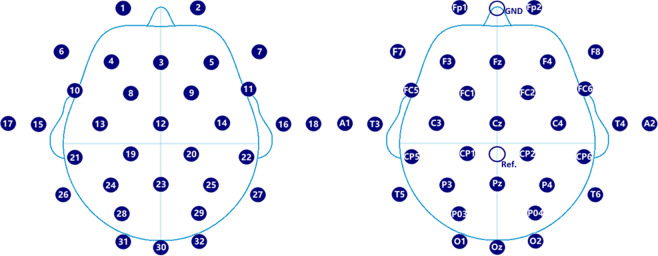
EEG cap electrode distribution.

The solid electrode cap with Ag/AgCl led by 32 (according to the standard 10–10 System, see in Fig. 2) from Wuhan Greentech Technology Co., LTD was selected. The electrode cap has the advantages of high current density, good anti-interference, and low impedance. The amplifier was a wireless amplifier manufactured by Brickcom, which supports wireless transceiver mode and real-time impedance. monitoring During the acquisition process, the electrode impedance was kept below 20 KΩ, and the sampling frequency was 250 Hz. Data was stored in the unit of uV. The EEG cap electrode distribution was shown in Fig. 2. Table 2 listed the detailed information of the dataset.

**Table 2:** Detailed information about the dataset.

Parameters	Values
Number of subjects	25
Number of classes	2
Number of sessions for each subject	5
Number of trials per session	90 to 100
Sampling rate	250 Hz
Sample resolution	24 bits
Number of channels	32

EEG bad segments were removed before preprocessing. The method of removing the bad segment was that EEGLAB automatically marked the amplitude more than 100uV. Then, the segments were judged comprehensively whether to be bad according to the visual observation of two researchers with rich BCI experience. 4 s EEG data of MI task was saved to facilitate MI algorithm processing. The sampling frequency was 250 Hz, and the total time samples of each trial were 1000. All data was removed the baseline, and 0.5–40 Hz band-pass filtering processing was carried out with a finite impulse response (FIR) filter before disclosure. The EEG data in some sessions was missing a small number of trials due to removing bad segments.

**Fig. 3:**
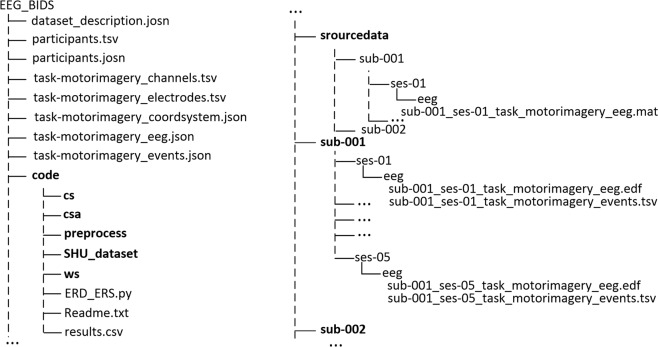
Directory tree for the repository with previews of EEG files. The tree on the right joins the end of the tree on the left.

The dataset was open access for free download at figshare^17^. The source files and EEG data files in this dataset were organized according to EEG-BIDS^28^, which was an extension of the brain imaging data structure for EEG. There were many ways to access data, such as IEEE P2731^29^, FAIR^30^, and EEG-BIDS. Among them, IEEE P2731 defines a complete storage system, which included decoding algorithms, preprocessing, feature extraction, and classification. The system fully described the process of generating, processing, and using EEG datasets. But most researchers were more concerned about the EEG data itself, especially the data that can be used directly without relying on a huge system. EEG-BIDS was particularly suitable for this storage requirement. It provided EEG data with relevant information files which record almost all the information covering the experiment, which can help users use these data quickly and directly. Meanwhile, EEG-BIDS was findable, accessible, interoperable, and reusable as FAIR storage rules require. The directory tree for our repository and some previews for meta-data were shown in Fig. 3. The dataset consisted of three parts: (1) Code, which provided the source code used in Technical Validation to help researchers quickly master how to use the dataset; (2) Processed data was the raw data after preprocessing, saved as the ‘.edf’ files, named according to the subject number and session number. ‘sub-xxx’ denoted the subject number and ‘ses’ denoted the session number; (3) Trial Data was directly available for classification and. The naming format of Trial Data was the same as (2).

## Data Records

### Processed data

The raw data was imported into MATLAB (http:// https://www.mathworks.com/) using the EEGLAB (http://sccn.ucsd.edu/eeglab) toolbox, and after manual preprocessing operations such as bad trials removal, baseline removal, and FIR filtering. The preprocessed data of each session was saved as a file (‘FILENAME.edf’, for float data). It is organized according to the following rules:

$$Sub\,\mbox{--}\,xxx\_ses\,\mbox{--}\,yy\_task\,\mbox{--}\,motorimagery\_eeg.edf$$

where ‘xxx’ was the subject number (001, 002, …, 025), ‘yy’ was the session number (01, 02, …, 05), and MI represents the task MI task. The information of channel names and channel locations were saved in ‘.edf’ files. ‘FILENAME.tsv’ provided the event information and event labels.

### Trial data

The EEG data for each subject of each session was saved as a ‘.mat’ file. The data for each subject (number: 001, 002,…, 025) was stored as a first-level directory. The ‘.mat’ files of the same subject in the same directory represented the 5 independent sessions of the same subject. The file naming rules were as follows.

$$Sub\,\mbox{--}\,xxx\_ses\,\mbox{--}\,yy\_task\,\mbox{--}\,motorimagery\_eeg.mat$$

where ‘xxx’ was the subject number (001, 002, …, 025), ‘yy’ was the session number (01, 02, …, 05), and ‘motor imagery’ was the task of MI.

‘.mat’ file contained two variables:

- data: 100 trials of MI data (A small number of trials in some sessions were removed due to exceptions). The data size was trial numbers, channel numbers, and time samples (100, 32, 1000).
- labels: It contains the task labels (“1” and “2” for MI of left-hand and right-hand) of the subject.

## Technical Validation

### Event-related desynchronization/synchronization

The EEG data of C3 and C4 from all subject channels were first band-pass–filtered with an 8–30 Hz FIR filter. Then calculated the Event-related desynchronization/synchronization (ERD/ERS) of the C3 and C4 channels as follows^5,31^. Calculate the square of each trial data. The C3 and C4 channel data of the same task were superimposed and averaged according to the number of trials. The average curve obtained was smoothed with a sliding time window. Equation 1 was used to calculate the EEG energy of each time sample. *x*_*c, i*_ was the amplitude of the EEG channel *c* at time sample *i*, *n* was the time samples ranged from 1 to 1000. Equation 2 was the smooth method, in which *P*_*c, i*_ was the energy value of the EEG channel *c* at the time sample *i*, *l* was the time window of smooth (value was 200 in this paper). Equation 3 was the ERD/ERS, where *Y*_*mean*_ was the average of the first 125 time samples *Y*_*c*_.

1 $${p}_{c,i}=\frac{1}{n}\mathop{\sum }\limits_{i=1}^{n}{x}_{c,i}^{2}$$

2 $${Y}_{c,n}=\frac{1}{l}\mathop{\sum }\limits_{i=n}^{n+l}{P}_{c,i}$$

3 $${E}_{c,n}=({Y}_{c,n}-{Y}_{mean})/{Y}_{mean}$$

**Fig. 4:**
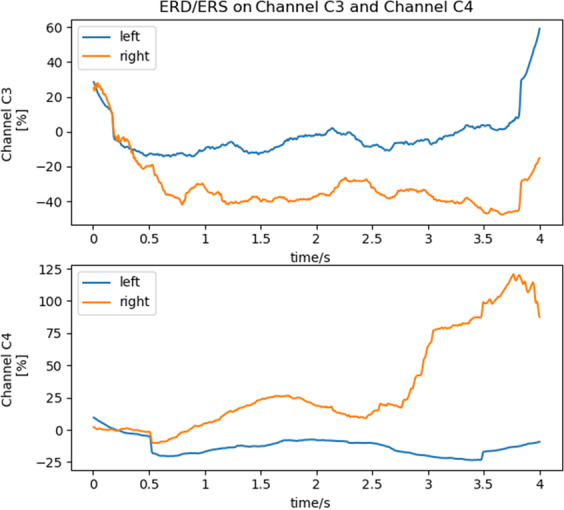
ERD/ERS results of C3 and C4 channels in left-hand and right-hand MI tasks.

Figure 4 showed the ERD/ERS of the EEG on the C3 and C4 channels of the left-hand and right-hand MI tasks. The results showed that the EEG signal energy of the left-hand MI task in the C3 channel was significantly higher than that of the right-hand. Similar results also appeared on the C4 channel. The ERD/ERS results verified the reliability of the dataset in the time domain.

### Within-session classification

In WS, all trials of each session were randomly divided into the training set, verification set, and test set respectively according to the proportion of 8:1:1. The result was the average accuracy of 10-fold cross-validation. This paper selected two classical algorithms (CSP and FBCSP), and three deep learning methods (FBCNet, deep ConvNets, and EEGNet) for classification.

CSP created an optimal spatial filter through supervised learning, which required minimizing the variance of another class when the variance of one class was maximum^12^. The CSP eigenmatrix *Z* = *WE* was obtained by diagonalizing and projecting the covariance matrix of the orthogonal whitening of two kinds of eigenmatrices simultaneously. *Z* was the spatially filtered signal, *W* was the characteristic matrix, and *E* was the EEG data of one trail. The dimension of the CSP feature matrix was the same as the channel number of the original data. The feature matrix’s first and last column of the feature matrix represent the maximum differentiation of the two classes. The CSP feature extraction configuration included a 3–35 Hz FIR band-pass filtering and a time interval of 0 to 4 s. The support vector machine (SVM) classifier was used to train and test. The first and last columns were picked according to the feature matrix of the spatial filter in CSP, which were considered the most effective spatial filtering features for the first (left hand) and last (right hand) EEG data. The values of the two-column feature matrix were then mapped onto the brain map to draw the CSP features with the values as the energy distribution of the brain map.

The FBCSP added a filter bank as a multi-band input based on CSP^19^. The algorithm divided the original signal into multiple subbands and extracted the CSP features of each subband to obtain filter-bank features. The configuration of the algorithm included a time interval of 0.5 to 4 s. The SVM classifier was used for training and testing the algorithm.

Three deep learning algorithms included FBCNet, EEGNet, and deep ConvNets. They were all based on convolutional neural networks. The three algorithms used different network structures, and FBCNet also used filter-bank data as input, the same as FBCSP. Three deep learning algorithms had unified training parameters: the batch size was 16, the learning rate was 0.001, maximum iteration times was 1500 epoch, loss function was ‘NLLLoss’, and optimizer was ‘Adam’. Training would stop when the classification accuracy on the validation set stopped improving. The preprocessed data were directly used in three deep learning algorithms.

**Fig. 5:**
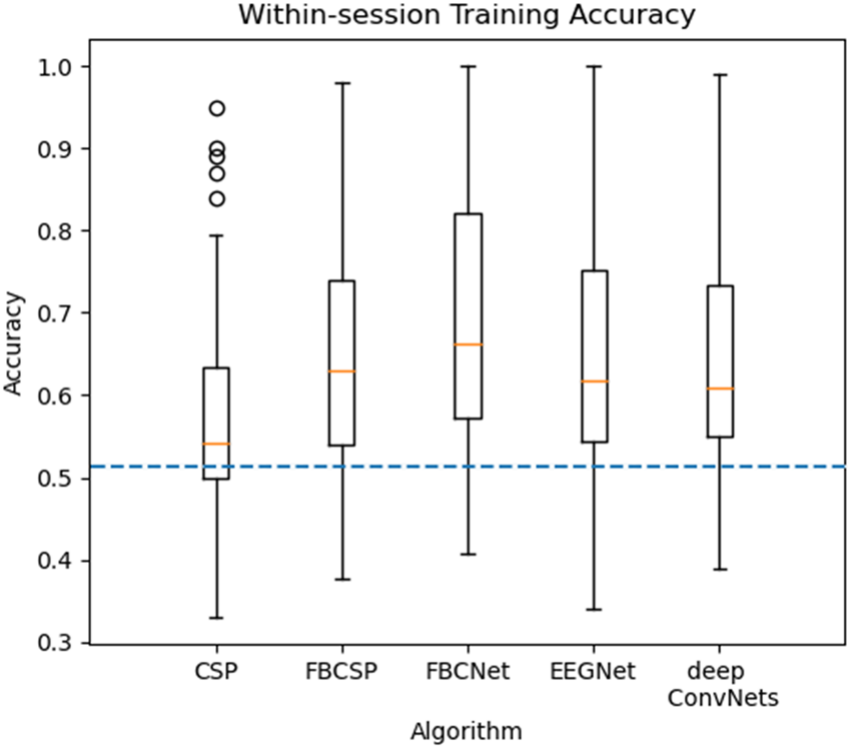
Classification accuracy of each algorithm of WS. The blue dash-dotted line indicates 51.4% accuracy chance level with p = 0.001 (N = 12500).

**Fig. 6:**
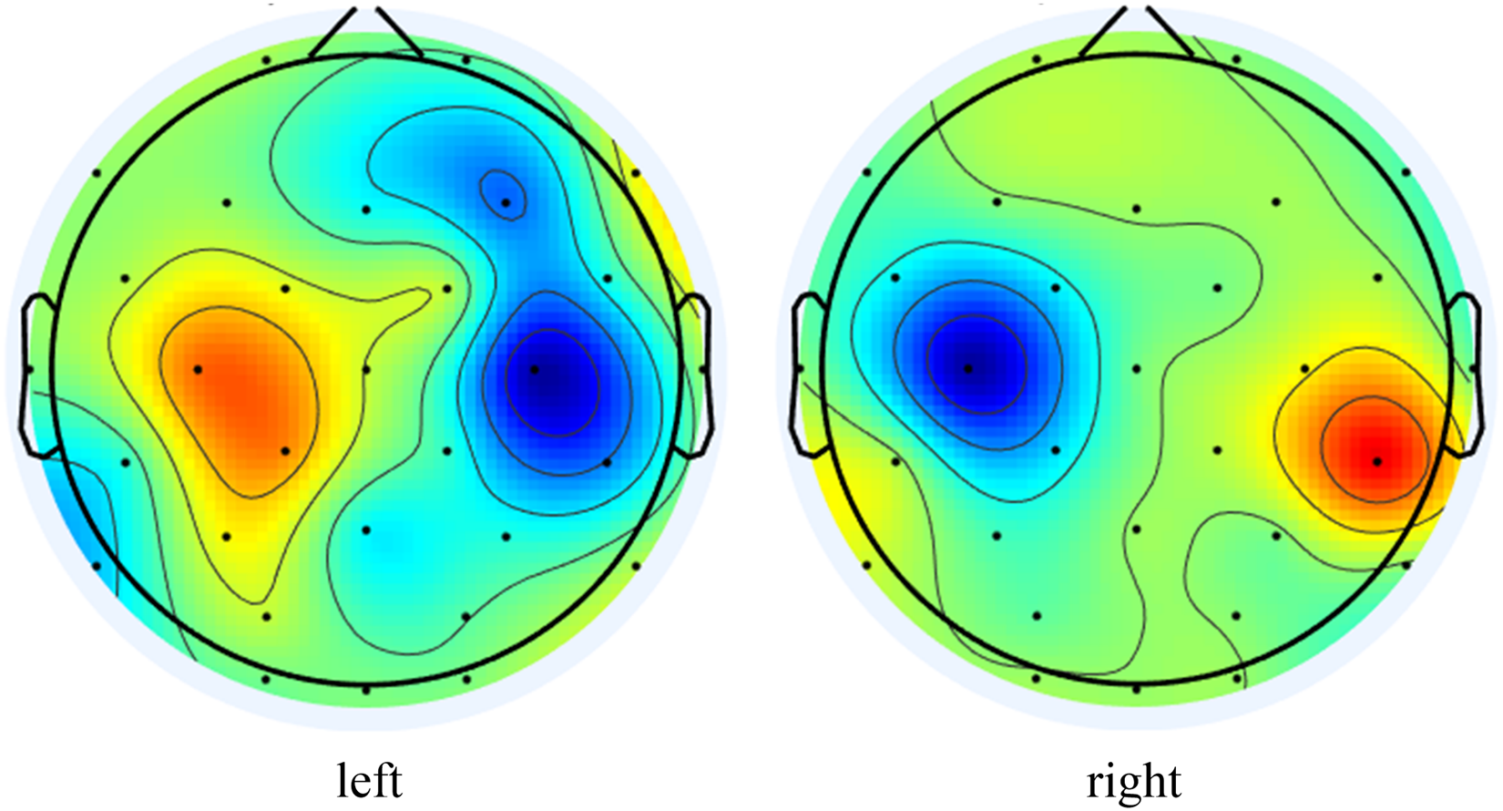
CSP feature brain map.

The average classification accuracy of the 125 independent sessions in the whole dataset modeled by the five WS algorithms was shown in Fig. 5. The average accuracy of FBCNet (accuracy was 68.8% ± 0.146) was the highest, indicating that the deep learning method based on the variance layer can better distinguish different tasks in this dataset. It can be seen that the filter bank of FBCSP and FBCNet got better results in feature extraction and classification. Compared to CSP, FBCSP, FBCNet, EEGNet, and deep ConvNets significantly (P < 0.001) improve classification accuracy. Through preliminary approaches, we determined the sufficient quality of our dataset. The samples with stable EEG amplitudes in all subjects with high classification performance were selected as input data, and the CSP was retrained. Thus the CSP feature brain map was obtained in Fig. 6.

### Cross-session classification

The three deep learning algorithms were the same in CS and WS. The difference was that the training set and the test set were changed. In CS, the data in the first session was used as the training set, and the data in the remaining four sessions of the same subject were used as the test set. Due to the difficulty of cross-session classification, the CSP and FBCSP algorithms were not used. The preprocessed data was used in three deep learning algorithms.

**Fig. 7:**
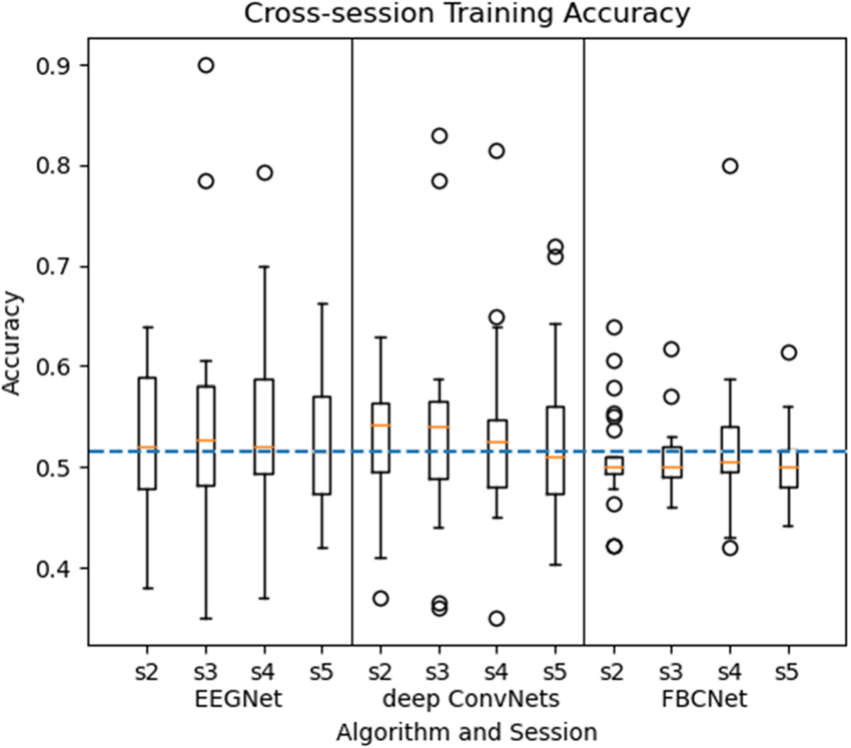
Classification accuracy of CS. EEGNet, deep ConvNets, and FBCNet represent the three deep learning algorithms. ‘s2’ to ‘s5’ represent different test sessions. The blue dash-dotted line indicates 51.6% accuracy chance level with p = 0.05 (N = 2500).

The classification accuracy of all 25 subjects in CS was shown in Fig. 7. The results showed that the performance of the three deep learning algorithms was not good in CS. However, the EEGNet algorithm in the case of CS was slightly better than the other two algorithms. 20 sessions with EEGNet classification accuracy were higher than 60%. Some subjects were still effectively distinguished, such as subjects No. 6, No. 13, No. 20, and No. 21. The classification accuracy of the third session from the No. 13 subject reached 90%, which was the highest among all CS classification results.

### Cross-session adaptation

The adaptive EEG^23^ was used in CSA, which added cross-subject migration adaptive algorithm based on deep ConvNets^22^ network structure. In CSA, one subject was selected as the target domain, and the rest of the subjects were selected as the source domain. The source domain was divided into a training set and a validation set. Randomly selected three subjects as the validation set and the rest as the training set. The learning rate of the base model in the source domain was set to 0.01. The target domain data was split into 90% training set and 10% test set according to 10-fold cross-validation. The learning rate of the base model was modified to 0.0005 and retrained using the target domain training set to obtain the classification model. Meanwhile, the adaptive training ratios of the target subject training set were 0%, 30%, 50%, 70%, 90%, and 100%.

**Fig. 8:**
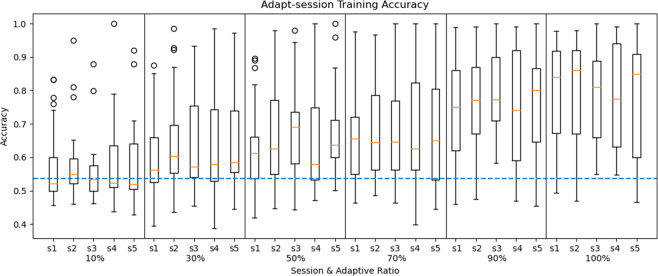
Classification accuracy of CSA. The horizontal axis represents the session number, and adaptive ratio. The blue dash-dotted line indicates 53.7% accuracy chance level with p = 0.001 (N = 2500).

The test results of CSA were shown in Fig. 8. We found that as the adaptive training set increased, the test set’s classification accuracy gradually increased. When the adaptive training set reached 50% (average accuracy was 70.52%), it was higher than the accuracy of the WS with a 90% training set (average accuracy was 68.8%). When the adaptive training set reached 100%, the correct rate of WS was improved by nearly 10% (average accuracy was 78.86%). Therefore, CSA had two advantages: reducing training samples and improving accuracy. Compared with CSP in WS, the classification accuracy of the adaptive training ratio above 50% improved significantly (P < 0.05). The improvement was very significant (P < 0.001) when the adaptive ratio was higher than 90%.

## Usage Notes

Users can directly use the codes to reproduce all the experimental results in the report. Three classification algorithms correspond to ‘ws’, ‘cs’, and ‘csa’ in the code folder. ‘ERD_ERS.py’ in the root directory was the ERD/S visualization algorithm. Before running the code, the user can create a ‘SHU_dataset’ folder in the code directory and copies all files in the ‘sourcedata’ to the ‘SHU_dataset’. The results of all algorithms were given in ‘results.csv’ in ‘code’ folder. All code run in python 3.7 (http://www.python.org). We provided the ‘mne’ data structure applicable to the python environment and the ‘EEGLAB’ data structure applicable to the MATLAB environment so that users can quickly choose their own algorithm scripts in need. The compressed packages were named after the file type, which contains all the content of this type of file, all uploaded to facilitate users to download data.

This dataset has multiple potential uses for cognitive neuroscience and for stroke rehabilitation development in EEG analysis, such as:

1. Within-session classification. One session data was split into a training set and a test set to evaluate the performance of the algorithm. The main purpose of WS was to improve the classification accuracy of traditional BCI rehabilitation training methods.
2. Cross-session classification. The data of the same subject for multiple sessions (up to five sessions) were selected as the training set for the previous one or more sessions to predict the next session (the test session data does not participate in the training session).
3. Cross-subject classification. The data of multiple subjects were transferred to train a better model to improve the classification accuracy. The main purpose of CSA was to study the optimal model using a minimum of target session data.

## Supplementary information

Supplementary Tables

## Data Availability

A script containing all the algorithms in this paper stored in ‘code.zip’ is provided with the dataset. All code is implemented in python (version python3.7 on Windows).
