# Supplementary material for: A large EEG dataset for studying cross-session variability in motor imagery brain-computer interface
Source: Sci Data. 2022 Sep 1;9:531. doi: 10.1038/s41597-022-01647-1 (PMC9436944; doi:10.1038/s41597-022-01647-1)
Supplement: Supplementary file 1 — Supplementary Tables [file 41597_2022_1647_MOESM1_ESM.pdf]

Supplementary Tables

Contents

Table 3: WS classification accuracy for all subjects, session 2
Table 4: CS classification accuracy for all subjects, session 3
Table 5a: CSA classification accuracy for all subjects, session 3
Table 5b: CSA classification accuracy for all subjects, session 4
Table 5c: CSA classification accuracy for all subjects, session 4

Table 3: WS classification accuracy for all subjects, session

Sub.	Session	CSP	FBCSP	FBCNet	EEGNet	deep ConvNets	Sub.	Session	CSP	FBCSP	FBCNet	EEGNet	deep ConvNets
1	1	0.550	0.730	0.780	0.570	0.600		4	0.537	0.499	0.672	0.778	0.728
	2	0.470	0.620	0.630	0.550	0.500		5	0.510	0.780	0.900	0.910	0.930
	3	0.566	0.688	0.678	0.617	0.568	14	1	0.500	0.560	0.670	0.620	0.620
	4	0.749	0.809	0.850	0.476	0.507		2	0.472	0.527	0.452	0.538	0.486
	5	0.587	0.689	0.707	0.529	0.447		3	0.547	0.567	0.642	0.556	0.600
2	1	0.738	0.871	0.761	0.870	0.860		4	0.423	0.502	0.458	0.739	0.619
	2	0.782	0.918	0.550	0.549	0.733		5	0.520	0.570	0.560	0.570	0.590
	3	0.600	0.510	0.610	0.550	0.520	15	1	0.540	0.420	0.540	0.510	0.510
	4	0.840	0.850	0.950	0.820	0.940		2	0.538	0.692	0.662	0.572	0.622
	5	0.758	0.830	0.829	0.751	0.824		3	0.485	0.376	0.407	0.518	0.551
3	1	0.500	0.567	0.600	0.540	0.636		4	0.538	0.692	0.662	0.572	0.622
	2	0.330	0.460	0.630	0.610	0.650		5	0.514	0.546	0.661	0.591	0.571
	3	0.544	0.558	0.478	0.556	0.568	16	1	0.760	0.640	0.820	0.630	0.540
	4	0.500	0.540	0.650	0.540	0.650		2	0.570	0.730	0.820	0.590	0.590
	5	0.560	0.540	0.690	0.730	0.600		3	0.610	0.650	0.570	0.630	0.610
4	1	0.433	0.649	0.483	0.549	0.567		4	0.683	0.798	0.800	0.483	0.619
	2	0.582	0.641	0.659	0.688	0.578		5	0.361	0.582	0.603	0.517	0.582
	3	0.539	0.553	0.571	0.639	0.660	17	1	0.640	0.760	0.820	0.760	0.790
	4	0.560	0.510	0.580	0.680	0.550		2	0.490	0.570	0.560	0.700	0.480
	5	0.590	0.770	0.780	0.540	0.790		3	0.527	0.546	0.661	0.591	0.571
5	1	0.522	0.620	0.542	0.548	0.532		4	0.760	0.640	0.820	0.630	0.540
	2	0.950	0.960	0.980	0.940	0.930		5	0.550	0.500	0.612	0.519	0.569
	3	0.570	0.694	0.580	0.493	0.561	18	1	0.513	0.481	0.614	0.534	0.604
	4	0.607	0.616	0.677	0.587	0.477		2	0.480	0.430	0.570	0.680	0.670
	5	0.517	0.463	0.528	0.472	0.563		3	0.390	0.580	0.740	0.680	0.660
6	1	0.616	0.894	0.990	0.864	0.919		4	0.570	0.730	0.820	0.590	0.590
	2	0.776	0.861	0.938	0.958	0.958		5	0.610	0.650	0.570	0.630	0.610
	3	0.692	0.822	0.916	0.801	0.822	19	1	0.626	0.571	0.699	0.589	0.636
	4	0.794	0.938	0.922	0.934	0.891		2	0.683	0.798	0.800	0.483	0.619
	5	0.674	0.752	0.877	0.844	0.843		3	0.694	0.791	0.884	0.830	0.851
7	1	0.640	0.610	0.600	0.590	0.580		4	0.489	0.548	0.597	0.584	0.609
	2	0.460	0.520	0.570	0.530	0.520		5	0.591	0.449	0.617	0.758	0.637
	3	0.519	0.576	0.573	0.560	0.499	20	1	0.694	0.899	0.931	0.933	0.900
	4	0.400	0.622	0.617	0.672	0.676		2	0.870	0.970	0.990	0.990	0.990
	5	0.500	0.770	0.800	0.850	0.760		3	0.460	0.560	0.540	0.490	0.560
8	1	0.530	0.680	0.700	0.770	0.750		4	0.900	0.970	1.000	1.000	0.990
	2	0.507	0.461	0.489	0.689	0.657		5	0.890	0.980	1.000	1.000	0.980
	3	0.518	0.588	0.483	0.900	0.889	21	1	0.720	0.742	0.891	0.937	0.926
	4	0.519	0.510	0.613	0.497	0.447		2	0.506	0.564	0.657	0.626	0.639
	5	0.480	0.460	0.570	0.540	0.480		3	0.632	0.550	0.851	0.763	0.796
9	1	0.461	0.634	0.554	0.602	0.511		4	0.790	0.670	0.920	0.930	0.920
	2	0.410	0.710	0.730	0.690	0.620		5	0.500	0.629	0.861	0.831	0.854
	3	0.490	0.480	0.630	0.450	0.530	22	1	0.463	0.545	0.575	0.510	0.408
	4	0.526	0.428	0.563	0.543	0.510		2	0.700	0.700	0.860	0.810	0.780
	5	0.574	0.739	0.789	0.634	0.634		3	0.530	0.540	0.530	0.620	0.540
10	1	0.550	0.563	0.617	0.540	0.585		4	0.650	0.840	0.920	0.930	0.900
	2	0.541	0.641	0.619	0.341	0.479		5	0.508	0.428	0.517	0.538	0.567
	3	0.680	0.890	0.690	0.640	0.630	23	1	0.636	0.689	0.648	0.779	0.717
	4	0.540	0.840	0.680	0.630	0.650		2	0.563	0.637	0.678	0.678	0.721
	5	0.634	0.741	0.634	0.567	0.579		3	0.596	0.624	0.828	0.809	0.798
11	1	0.530	0.590	0.590	0.650	0.600		4	0.610	0.750	0.770	0.820	0.850
	2	0.431	0.448	0.422	0.656	0.691		5	0.504	0.653	0.677	0.690	0.782
	3	0.510	0.640	0.630	0.640	0.620	24	1	0.457	0.450	0.484	0.564	0.521
	4	0.500	0.730	0.690	0.620	0.560		2	0.535	0.493	0.668	0.561	0.619
	5	0.620	0.710	0.780	0.560	0.520		3	0.465	0.584	0.661	0.679	0.604
12	1	0.360	0.480	0.580	0.470	0.590		4	0.491	0.642	0.714	0.583	0.636
	2	0.331	0.540	0.562	0.450	0.526		5	0.530	0.600	0.520	0.620	0.640
	3	0.512	0.580	0.502	0.507	0.444	25	1	0.536	0.537	0.589	0.526	0.479
	4	0.542	0.477	0.597	0.613	0.518		2	0.638	0.716	0.891	0.563	0.584
	5	0.431	0.427	0.506	0.492	0.493		3	0.627	0.776	0.882	0.478	0.492
13	1	0.590	0.730	0.840	0.800	0.750		4	0.738	0.691	0.726	0.499	0.476
	2	0.469	0.427	0.552	0.652	0.672		5	0.670	0.780	0.880	0.570	0.390
	3	0.730	0.860	0.930	0.940	0.920	Average		0.573	0.643	0.688	0.650	0.648

Table 4: CS classification accuracy for all subjects, session

Subjects/	EEGNet				deep ConvNets				FBCNet			
Session	2	3	4	5	2	3	4	5	2	3	4	5
1	0.470	0.465	0.566	0.500	0.460	0.515	0.505	0.404	0.480	0.495	0.505	0.532
2	0.579	0.480	0.630	0.663	0.558	0.440	0.540	0.621	0.579	0.460	0.500	0.495
3	0.520	0.554	0.630	0.480	0.590	0.489	0.540	0.500	0.500	0.522	0.500	0.480
4	0.629	0.527	0.510	0.530	0.505	0.549	0.460	0.550	0.423	0.505	0.420	0.500
5	0.590	0.441	0.525	0.438	0.410	0.452	0.556	0.438	0.510	0.527	0.525	0.517
6	0.628	0.484	0.793	0.663	0.585	0.453	0.815	0.642	0.511	0.495	0.587	0.484
7	0.500	0.573	0.587	0.570	0.490	0.573	0.554	0.510	0.500	0.521	0.543	0.560
8	0.500	0.506	0.490	0.450	0.553	0.551	0.469	0.510	0.479	0.618	0.500	0.510
9	0.480	0.530	0.521	0.525	0.520	0.520	0.500	0.515	0.500	0.480	0.521	0.465
10	0.479	0.460	0.370	0.515	0.585	0.450	0.350	0.485	0.500	0.570	0.500	0.515
11	0.489	0.590	0.510	0.610	0.553	0.580	0.450	0.580	0.479	0.490	0.480	0.500
12	0.462	0.536	0.494	0.470	0.505	0.546	0.494	0.434	0.495	0.485	0.540	0.470
13	0.617	0.900	0.453	0.560	0.564	0.830	0.547	0.710	0.553	0.530	0.505	0.530
14	0.453	0.598	0.522	0.480	0.453	0.587	0.478	0.510	0.421	0.467	0.478	0.460
15	0.589	0.482	0.526	0.643	0.547	0.365	0.537	0.469	0.463	0.494	0.463	0.510
16	0.550	0.580	0.516	0.571	0.530	0.540	0.526	0.560	0.550	0.500	0.495	0.473
17	0.380	0.551	0.520	0.453	0.370	0.582	0.590	0.484	0.500	0.500	0.530	0.442
18	0.520	0.350	0.450	0.510	0.550	0.360	0.450	0.440	0.500	0.490	0.540	0.490
19	0.621	0.521	0.533	0.473	0.495	0.553	0.533	0.613	0.495	0.521	0.489	0.473
20	0.400	0.480	0.700	0.520	0.580	0.550	0.640	0.460	0.640	0.480	0.800	0.540
21	0.586	0.785	0.700	0.615	0.566	0.785	0.650	0.719	0.606	0.495	0.540	0.615
22	0.640	0.490	0.520	0.516	0.630	0.510	0.520	0.473	0.500	0.500	0.430	0.484
23	0.457	0.606	0.630	0.608	0.543	0.535	0.520	0.546	0.511	0.525	0.550	0.495
24	0.549	0.590	0.480	0.420	0.451	0.566	0.480	0.480	0.537	0.482	0.520	0.520
25	0.489	0.500	0.466	0.470	0.522	0.500	0.545	0.520	0.511	0.511	0.568	0.500
Average	0.527	0.543	0.546	0.530	0.525	0.535	0.530	0.527	0.510	0.506	0.521	0.502

Table 5a: CSA classification accuracy for all subjects, session

Subject/	0% adaptation					30% adaptation				
Session	1	2	3	4	5	1	2	3	4	5
1	0.600	0.510	0.576	0.474	0.520	0.600	0.630	0.566	0.544	0.527
2	0.500	0.538	0.500	0.510	0.621	0.490	0.600	0.550	0.610	0.747
3	0.543	0.490	0.543	0.520	0.510	0.683	0.690	0.489	0.590	0.620
4	0.564	0.609	0.593	0.580	0.470	0.544	0.617	0.647	0.630	0.510
5	0.522	0.540	0.471	0.516	0.429	0.454	0.940	0.609	0.578	0.539
6	0.778	0.652	0.486	0.748	0.684	0.832	0.907	0.840	0.922	0.822
7	0.490	0.460	0.520	0.533	0.580	0.600	0.510	0.460	0.476	0.740
8	0.530	0.562	0.607	0.520	0.500	0.690	0.657	0.811	0.510	0.490
9	0.500	0.540	0.530	0.523	0.446	0.612	0.530	0.540	0.532	0.504
10	0.496	0.487	0.490	0.500	0.452	0.494	0.477	0.650	0.550	0.429
11	0.500	0.522	0.540	0.520	0.520	0.500	0.586	0.550	0.540	0.570
12	0.600	0.560	0.536	0.514	0.515	0.660	0.582	0.494	0.563	0.557
13	0.520	0.533	0.880	0.636	0.880	0.860	0.586	0.950	0.746	0.970
14	0.500	0.517	0.518	0.652	0.640	0.600	0.511	0.596	0.598	0.700
15	0.500	0.506	0.463	0.506	0.511	0.600	0.548	0.411	0.548	0.592
16	0.490	0.530	0.500	0.524	0.517	0.450	0.630	0.760	0.554	0.638
17	0.760	0.550	0.521	0.540	0.506	0.720	0.580	0.610	0.570	0.640
18	0.489	0.780	0.540	0.690	0.710	0.484	0.810	0.700	0.720	0.780
19	0.602	0.587	0.510	0.543	0.606	0.689	0.714	0.819	0.682	0.757
20	0.833	0.950	0.500	1.000	0.920	0.856	0.990	0.470	0.990	0.980
21	0.832	0.574	0.557	0.790	0.592	0.892	0.668	0.828	0.840	0.853
22	0.506	0.610	0.610	0.780	0.664	0.593	0.890	0.600	0.930	0.592
23	0.741	0.811	0.799	0.500	0.702	0.789	0.800	0.806	0.920	0.794
24	0.543	0.596	0.600	0.438	0.490	0.584	0.681	0.660	0.593	0.560
25	0.457	0.587	0.541	0.475	0.520	0.579	0.542	0.564	0.503	0.480
Average	0.576	0.584	0.557	0.581	0.580	0.634	0.667	0.639	0.650	0.656

Table 5b: CSA classification accuracy for all subjects, session

Subject/	50% adaptation					70% adaptation				
Session	1	2	3	4	5	1	2	3	4	5
1	0.570	0.520	0.757	0.607	0.514	0.570	0.500	0.807	0.557	0.556
2	0.816	0.806	0.710	0.770	0.728	0.654	0.569	0.710	0.850	0.924
3	0.607	0.790	0.511	0.570	0.610	0.748	0.590	0.684	0.640	0.660
4	0.553	0.718	0.659	0.710	0.710	0.507	0.734	0.560	0.750	0.630
5	0.546	0.930	0.564	0.556	0.597	0.467	0.950	0.592	0.606	0.763
6	0.906	0.968	0.914	0.889	0.903	0.819	0.968	0.958	0.846	0.936
7	0.560	0.570	0.491	0.543	0.800	0.550	0.730	0.584	0.452	0.820
8	0.690	0.720	0.899	0.406	0.490	0.720	0.650	0.943	0.550	0.520
9	0.660	0.770	0.580	0.640	0.754	0.638	0.880	0.800	0.521	0.827
10	0.626	0.589	0.670	0.660	0.668	0.671	0.602	0.760	0.770	0.402
11	0.650	0.770	0.680	0.550	0.670	0.680	0.619	0.810	0.770	0.580
12	0.630	0.604	0.606	0.560	0.683	0.730	0.604	0.693	0.564	0.719
13	0.850	0.596	0.990	0.829	0.940	0.840	0.667	0.970	0.758	0.970
14	0.670	0.461	0.740	0.619	0.780	0.560	0.681	0.771	0.851	0.710
15	0.650	0.588	0.490	0.578	0.653	0.620	0.620	0.565	0.640	0.582
16	0.700	0.660	0.770	0.596	0.628	0.640	0.670	0.730	0.660	0.780
17	0.820	0.610	0.642	0.620	0.662	0.860	0.650	0.659	0.720	0.659
18	0.601	0.770	0.800	0.850	0.840	0.751	0.800	0.690	0.850	0.850
19	0.741	0.683	0.883	0.750	0.676	0.634	0.706	0.947	0.761	0.809
20	0.807	0.990	0.530	1.000	1.000	0.789	0.990	0.680	0.990	1.000
21	0.881	0.678	0.859	0.880	0.861	0.936	0.658	0.924	0.910	0.914
22	0.704	0.860	0.700	0.950	0.618	0.446	0.910	0.730	0.960	0.613
23	0.813	0.852	0.847	0.930	0.844	0.883	0.853	0.837	0.910	0.886
24	0.575	0.722	0.758	0.683	0.570	0.727	0.685	0.774	0.632	0.530
25	0.668	0.698	0.643	0.665	0.620	0.825	0.630	0.636	0.660	0.700
Average	0.692	0.717	0.708	0.696	0.713	0.691	0.717	0.753	0.727	0.734

Table 5c: CSA classification accuracy for all subjects, session

Subject/	90% adaptation					100% adaptation				
Session	1	2	3	4	5	1	2	3	4	5
1	0.580	0.710	0.908	0.517	0.820	0.580	0.690	0.888	0.776	0.568
2	0.970	0.603	0.750	0.930	0.927	0.970	0.558	0.810	0.930	0.970
3	0.468	0.640	0.719	0.590	0.650	0.740	0.930	0.723	0.580	0.920
4	0.851	0.660	0.724	0.550	0.920	0.831	0.926	0.571	0.940	0.900
5	0.488	0.980	0.783	0.666	0.829	0.846	0.970	0.809	0.666	0.908
6	0.949	0.958	0.958	0.967	0.958	0.949	0.883	0.874	0.978	0.969
7	0.840	0.870	0.698	0.676	0.840	0.860	0.470	0.659	0.774	0.850
8	0.860	0.622	0.833	0.470	0.690	0.770	0.778	0.978	0.553	0.540
9	0.722	0.700	0.850	0.532	0.646	0.673	0.920	0.860	0.602	0.536
10	0.738	0.476	0.750	0.500	0.454	0.864	0.704	0.750	0.770	0.466
11	0.780	0.872	0.930	0.590	0.550	0.940	0.916	0.700	0.900	0.910
12	0.620	0.771	0.772	0.750	0.760	0.770	0.762	0.556	0.621	0.807
13	0.880	0.892	1.000	0.938	1.000	0.840	0.607	1.000	0.960	1.000
14	0.750	0.607	0.694	0.773	0.790	0.850	0.937	0.907	0.786	0.690
15	0.620	0.673	0.593	0.694	0.683	0.620	0.557	0.588	0.548	0.591
16	0.460	0.670	0.660	0.831	0.802	0.570	0.880	0.780	0.704	0.833
17	0.810	0.680	0.588	0.610	0.641	0.920	0.670	0.623	0.610	0.800
18	0.692	0.870	0.900	0.800	0.850	0.844	0.860	0.870	0.930	0.880
19	0.729	0.706	0.980	0.764	0.618	0.833	0.909	0.980	0.631	0.909
20	0.989	0.990	0.710	0.990	1.000	0.978	0.980	0.550	0.990	1.000
21	0.904	0.856	0.904	0.920	0.916	0.970	0.709	0.980	0.980	0.874
22	0.658	0.810	0.810	0.940	0.581	0.493	0.950	0.850	0.990	0.809
23	0.867	0.877	0.858	0.950	0.866	0.918	0.877	0.828	0.940	0.908
24	0.605	0.810	0.757	0.742	0.800	0.620	0.644	0.910	0.727	0.600
25	0.793	0.807	0.583	0.479	0.560	0.526	0.532	0.593	0.921	0.510
Average	0.745	0.764	0.789	0.727	0.766	0.791	0.785	0.785	0.792	0.790
